# Prevalence and Associated Factors of Subclinical Mastitis Among Dairy Cows in Ethiopia: A Systematic Review and Meta-Analysis

**DOI:** 10.1155/vmi/2401778

**Published:** 2025-04-02

**Authors:** Tsegaye Asredie Kolech, Abebe Belete Bitew, Sefinew Alemu Mekonnen

**Affiliations:** ^1^Gondar Agricultural Research Center, P.O. Box 1337, Gondar, Ethiopia; ^2^Department of Veterinary Epidemiology and Public Health, College of Veterinary Medicine and Animal Sciences, University of Gondar, P.O. Box 196, Gondar, Ethiopia

**Keywords:** dairy cows, Ethiopia, meta-analysis, pooled prevalence, subclinical mastitis, systematic review

## Abstract

Subclinical mastitis (SCM) is an inflammation of the mammary glands without visible changes on milk or under. In dairy cattle production, it is the common and economically significant form of mastitis. Despite such impacts, little is known about its prevalence and associated factors in the different regions of Ethiopia. Hence, this review aimed to estimate the pooled prevalence of SCM from studies reported on Ethiopian dairy cows and explore factors associated with the prevalence of SCM. Articles reporting SCM in the Ethiopian dairy cows, published between 2012 and 2022, were searched from EMBASE, PubMed, Science Direct, Scopus databases, and Google scholar. Article identification, screening, and inclusion were made following the Preferred Reporting Items for Systematic Reviews and Meta-Analyses (PRISMA) guideline. Data were extracted independently and reviewed by two reviewers, and the trim-and-fill method was used to assess publication bias between studies. Data were managed using statistical tools in … software (Version …). Thirty-four eligible cross-sectional studies were included in the systematic review and meta-analysis. The pooled prevalence of SCM in Ethiopian dairy cows was found to be 43.19% (95% CI: 38.24%–48.13%). It was found that the prevalence of SCM varied between studies (*I*^2^ = 97.12%; *p* < 0.001). Based on subgroup and meta-regression analyses, Addis Ababa had the highest estimated prevalence of SCM at 54.11% (95% CI: 40.18–68.03), followed by Amhara region at 52.07% (95% CI: 34.49–69.66). The review revealed that SCM is prevalent in Ethiopian dairy cows, with different factors associated with its prevalence. To ensure dairy cows' welfare as well minimize the public health risks from the milk, early detection and proper management of SCM would be crucial.

## 1. Introduction

Mastitis is one of the most common diseases in dairy cows in Ethiopia, which significantly impacts animal health and the dairy industry [1]. It can manifest in either subclinical or clinical forms, with both forms having serious economic and health consequences [2]. The subclinical mastitis (SCM) is particularly insidious as it shows no obvious symptoms but results in a high somatic cell count (SCC) of more than 200,000 cells/mL. This reduces milk production, lowers reproductive success, and increases animal mortality [3]. In addition, SCM is an important risk factor for the development of clinical mastitis (CM), which can spread rapidly in dairy herds and sometimes requires the culling of affected animals [4].

The primary reported bacterial pathogens responsible for mastitis in Ethiopia include *Escherichia coli*, *Streptococcus agalactiae*, and *Staphylococcus aureus* [5], with *Staphylococcus* and *Streptococcus* being particularly common causes of SCM [2]. Despite the widespread prevalence of the disease, there is a lack of comprehensive awareness among Ethiopian dairy producers about the screening and detection of SCM and the hidden productivity losses associated with it [6]. This knowledge gap makes it difficult to effectively combat the disease.

Globally, SCM is a major problem for the dairy industry, particularly in developing countries where intensive farming systems have led to structural changes that exacerbate the prevalence of the disease [7]. In Ethiopia, numerous studies have reported the widespread occurrence of SCM, especially in the northwestern and central regions [6, 8–10]. However, there is still a lack of detailed information on the risk factors and pathogens contributing to the spread of SCM in the country. The economic losses associated with SCM, particularly regarding reduced milk yield, are significant. Production losses in crossbred cows due to SCM are estimated to be upto USD 38 per cow per lactation [11].

A meta-analysis, a growing but under-researched method in animal and veterinary science, systematically summarizes the results of multiple studies to draw solid conclusions based on a larger body of evidence [12]. This approach is increasingly valuable for decision-making in animal health and agriculture [1]. Several meta-analyses have focused on SCM in Ethiopia [1, 13, 14] and have revealed important insights into the prevalence of SCM and associated factors. However, there are still gaps in the existing literature. These include incomplete coverage of recent studies, inconsistent methodologies, and insufficient exploration of new factors such as the association of changing agricultural practices, crossbreeding, and socioeconomic challenges with the prevalence of SCM in Ethiopia. Given the importance of SCM in the Ethiopian dairy industry, this systematic review and meta-analysis aimed to pool the prevalence of SCM in dairy cows in Ethiopia from 2012 to 2022 and identify potential factors associated with the pooled prevalence of SCM.

## 2. Methods

### 2.1. Search Strategy

A preliminary search was conducted to identify relevant articles, confirm the validity of the meta-analysis, prevent the repetition of previously answered questions, and ensure that there were sufficient publications for this study [15]. The 2020 PRISMA expanded checklist was followed in the identification, screening, and inclusion of studies [16].

The keywords from the research question, bacteriologically verified SCM in Ethiopian dairy cows, were used to create a search strategy. Appropriate Medical Subject Headings (MeSH) terms/phrases and free text words were generated for each main idea, including the keywords of SCM, surveillance, prevalence, epidemiologic study, cattle, dairy cows, or dairy heifers. Each study report that quantified and summarized the association with SCM was included. Published articles were searched from PubMed, Science Direct, Scopus, EMBASE databases, and Google Scholar. To combine the MeSH terms either separately or in the proper combination, Boolean logical operators like “AND” and “OR” were employed.

### 2.2. Eligibility Criteria

Peer-reviewed published articles with freely accessible full text, written in English, and published between January 2012 and September 2022 were included. Cross-sectional studies on SCM that described the prevalence and associated factors of SCM in dairy cows from Ethiopia were considered. Reports that were not included were conference papers, case reports, journals without full access, articles mainly on CM in dairy cows, reports without abstracts, and systematic reviews and meta-analyses. Moreover, reviews, reports on other animals (such as sheep, goats, and camels) and studies on dairy cows with a sample size of less than 40 cows were excluded.

### 2.3. Literature Searching

**Figure 1:**
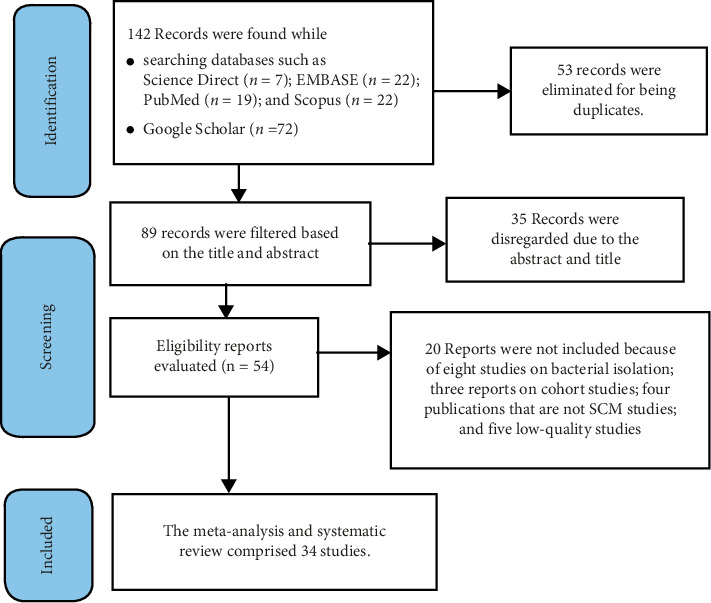
Flow diagram of identification literature and screening.

After removing duplicates, all citations found using our search approach were exported to the reference management program EndNote Version 20. Two independent authors assessed all eligible study reports based only on their titles and abstracts; studies that did not meet the eligibility requirements were screened out. Before data extraction, the full texts of the selected articles were retrieved and carefully reviewed to ensure whether acceptable. To include research that was missed by the search method, a manual search of the reference lists of some selected articles was performed. A 2020 PRISMA flowchart (Figure 1) was used to illustrate study selection procedures.

### 2.4. Data Extraction Process

Two independent authors extracted the data using a standard data collection form designed based on the key variables required for the primary research. Any disagreements between the two authors regarding data extraction were resolved by an impartial third author. The data extraction included variables such as primary authorship, publication year, sample size, prevalence rate, study design, study region, SCM risk factors, measures of association, and prevalence of SCM. The selected studies were reviewed multiple times, and qualitative and quantitative data were extracted using a pre-established data collection template.

### 2.5. Study Quality Assessment

Three people carried out the quality assessments using the Joana Briggs Institute(JBI) appraisal tool for the cross-sectional studies checklist [17]. An instrument of eight questions was utilized for articles that featured basic cross-sectional investigations. The tool comprises questions with a “yes” or “no” response option; answers were scored as 1 for “yes” and 0 for “no.” The results were added up and expressed as a percentage. Only studies with a score of ≥ 5 were considered for the systematic review and meta-analysis [18]. The causes of any discrepancies in scores between the extractors and the abstractors were investigated and all issues were resolved by revision.

### 2.6. Statistical Analysis

Statistical tools of Stata Version 17.0 (Stata Corp) were used to analyze the data that were retrieved. Data collected from pertinent research and presented in the evidence tables were summarized using descriptive statistics. By using meta-analysis, the pooled prevalence of SCM was determined. For the meta-analysis of proportions, the effect measure was computed using the Meta function of Stata. To investigate the causes of heterogeneity, meta-regression and subgroup analyses were performed. We expected heterogeneity and used the I^2^ and Cochrane's Q statistics (represented as *χ*^2^ and *p* values) to assess the differences. According to the I^2^ value, heterogeneity is categorized as low at 25%, moderate at 50%, and high at 75% [19]. A subgroup analysis was conducted to further investigate potential sources of heterogeneity. A forest plot was created to display the individual and pooled prevalence of SCM with 95% CI, the author's name, the year of publication, and study weights (for both primary studies and the systematic review/meta-analysis). Egger's test, the funnel plot, and “Duval and Tweedie's Trim and Fill” statistics were used to check for publication bias between studies. The extracted data were transformed into logits to ensure proper representation of study points in the funnel plot.

## 3. Results

### 3.1. Literature Search Results

A total of 142 SCM-related research articles were identified through the initial database search. Of these, 35 records were excluded based on abstract and title, and duplicates were removed. After a full-text review, 54 articles were considered. After removing 20 articles for various reasons, 34 eligible articles were included in the systematic review and meta-analysis. The JBI critical appraisal checklist for analytical cross-sectional studies was used to evaluate each study's quality. Based on the established criteria, 34 full-text articles with a high-quality score (≥ 5) were included in the data abstraction. The details of the literature search, identification, screening, and inclusion process are shown in Figure 1.

### 3.2. Descriptive Characteristics of the Included Studies

The 34 articles were found assessing 12,073 animals in total. About 19 studies were retrieved from the regional state of Oromia. Southern Nations, Nationalities, and People (SNNP) regional states contributed five published articles. Three articles from each were published by each of the regional states of Tigray, Amhara, and Addis Ababa (AA). One study was conducted in the Benishangul Gumuz (BG) region.  Table 1 outlines the features of every survey that qualifies.

### 3.3. Pooled Prevalence of SCM

**Figure 2:**
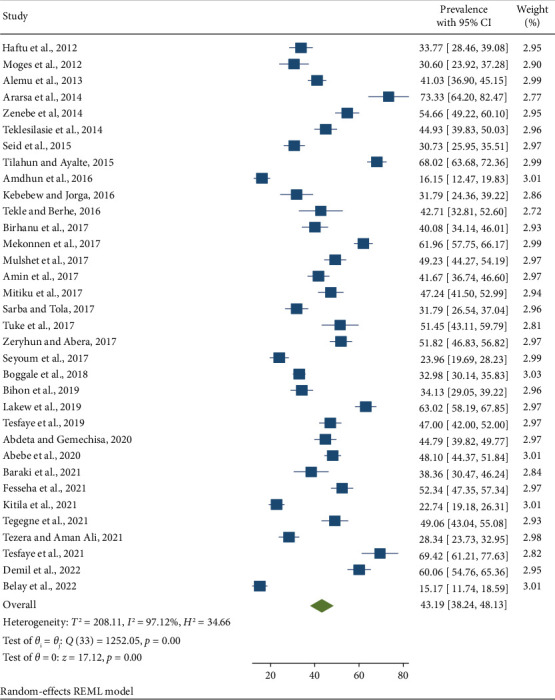
Forest plot showing the prevalence of SCM in Ethiopian dairy cows from 2012 to 2022.

Using a random-effects model, the pooled prevalence of SCM in dairy cows from 2012 to 2022 was calculated to be 43.19% (95% CI: 38.24–48.13) (Figure 2). Significant heterogeneity between the studies was observed in the pooled prevalence of SCM in dairy cows (*I*^2^ = 97.12%; *p* < 0.001).

### 3.4. Subgroup Analysis of SCM

Focusing on study location, publication year, and sample size, subgroup analysis was conducted because the meta-analysis showed a potential source of heterogeneity (Table 2). It was found that a higher pooled prevalence of SCM (54.11%; 95% CI: 40.18–68.03) was found in AA compared with the regional states of Ethiopia (Table 2). The second highest pooled prevalence of SCM in dairy cows was 52.07% (95 CI: 34.49–69.66) in the Amhara regional state. A total of 20 studies reported 6288 samples from 2012 to 2017 and 14 study articles with 5785 samples from 2018 to 2022. The meta-analysis showed that from 2012 to 2017, the pooled prevalence of SCM was 43.25% (95% CI: 36.90–49.59), whereas for 2018 to 2022, it was 43.11% (95% CI: 34.96–51.27). Compared with the 384 and 390–1049 sample size categories, the pooled prevalence of SCM in the 90–383 sample size category was 44% (95% CI: 38.16–49.84).

### 3.5. Assessment of Publication Bias

**Figure 3:**
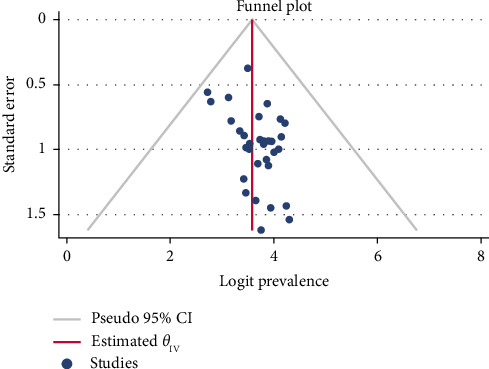
Funnel plot with pseudo 95% confidence interval limits for the analysis of publication bias.

**Figure 4:**
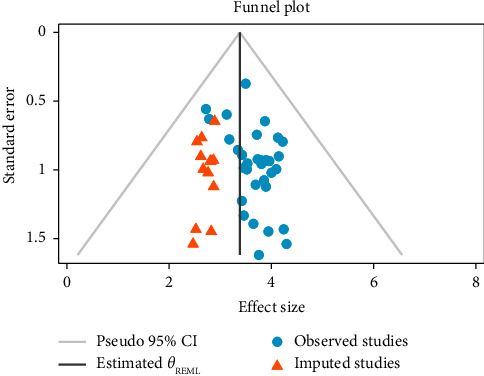
Trim and fill funnel plot that estimated the number of missing studies.

The presence of publication bias was assessed using a funnel plot and Egger's test, while Duval and Tweedie's Trim and Fill technique was used to obtain symmetry (Figures 3 and 4 and Table 3). Each point in the funnel plot was observed to be asymmetrical (Figure 3). Furthermore, Egger's test showed the possibility of publication bias (*p*=0.018), as shown in Table 3. Twelve studies were imputed using the “Duval and Tweedie's Trim and Fill” technique to obtain symmetry. Triangle dots represent studies inferred by the trim and fill process. The dots in Figure 4 reflect the initial logit prevalence estimate.

### 3.6. Factors Associated With the Pooled Prevalence of SCM

The pooled prevalence of SCM in dairy cows was associated with old age (*p*=0.001), parity (*p*=0.001), late lactation stage (*p*=0.004), and breed (*p*=0.046), as indicated in Table 4. The management system of the dairy cows was found to have no statistically significant association with the pooled prevalence of SCM (*p* > 0.05). The estimated pooled prevalence of SCM was 49% in crossbred breeds and 32% in indigenous breeds. Compared with adult and younger animals, the combined prevalence of SCM was significantly higher in older cows. Parity had a statistically significant association with the pooled prevalence of SCM, as seen by the higher frequency of 88% of cows with > 7 parities compared with those with parities 4–7 (54%) and 1–3. Compared with cows in the early and mid phases of lactation, those in the late stages of lactation experienced greater SCM symptoms.

## 4. Discussion

The pooled prevalence of SCM in this systematic review and meta-analysis was found to be 43.19%. In Ethiopia, this result agrees well with the prevalence of 43.6% reported in the meta-analysis by Girma and Tamir [13]. However, it is slightly lower than the 47% reported by Getaneh and Gebremedhin [1]. The current meta-analysis, which covers studies from 2012 to 2022, showed that the prevalence of SCM varies across regions. The current pooled prevalence of 43.19% was lower compared with the 46.35% reported in the systematic review and meta-analysis by Bangar et al. [48] in India and 48.2% by Khasapane et al. [14] in Africa. In contrast, it was higher than the 37.7% prevalence observed by Chen et al. [49] in China. Regional variations in farming methods and disease control techniques may have an impact on these disparities in prevalence rates. It has been demonstrated that several factors, including dry cow therapy, teat dipping, sanitary milking, appropriate management practices, and timely therapeutic interventions, are essential in lowering the prevalence of SCM [50].

According to the geographical administrative regional state distribution, SCM was most prevalent in AA (54.11%), followed by the Amhara region (52.07%). Owing to its poor state, Ethiopia produced a higher number of research among African nations, indicating that mastitis is a significant issue for dairy cows [51]. State-by-state meta-analysis aims to update the approach to the disease control and could give information about the prevalence of SCM in dairy cows in each state [48].

The current findings demonstrated that the estimations of SCM prevalence for both the overall analysis and specific regional states varied widely between studies. Agroclimatic considerations, farm management techniques, and variations in cow-level characteristics (genetic makeup of crossbreds, parity, and lactation stage) could all contribute to the variation in cow-level prevalence [52]. The prevalence of SCM was significantly associated with age, parity, stage of lactation, and breed. Within the age category of animals, older cows had a statistically significant (*p* < 0.05) higher pooled prevalence of SCM than younger and adult cows. Older cows may be more likely to have environmental and skin infections entering the teat canal [53]. Due to their more dilated and partially or permanently exposed teat canals, older animals may be more susceptible to SCM infection than younger animals [54]. Older cows had a higher prevalence, which may be related to their weakened teat canals, which make it easier for bacteria to invade the mammary gland after milking. In addition, older cows are more likely than younger cows to have pendulous udders, which are more vulnerable to damage and pathogen entrance. This may make older cows more susceptible to mastitis [55].

The findings showed an increase in SCM with an increase in the number of parties. According to Kayesh, Talukder, and Anower [56], the highest frequency was 88% in cows with a parity greater than seven. The greater frequency in cows with more than three parties may be the result of poor cow immunity or treatment-resistant mastitis-causing bacteria brought on by the careless use of antibiotics to treat mastitis in earlier parities or lactations [57]. Several research on the SCM risk factors [58, 59], such as those that have concentrated on small-scale farmers [60], repeatedly shown that multiparous cows are more likely than primiparous cows to get mastitis. The pooled prevalence of SCM in dairy cows increased along with cow parity. Given that longer milking cows have been exposed to SCM at a higher rate than cows that are just beginning to lactate, this could be explained.

The prevalence of SCM was higher in exotic zebu breeds than in Ethiopian local breeds. Studies have examined breed differences in dairy cattle's vulnerability to SCM [1, 13, 14, 48, 61, 62]. The genetic composition of the keratin and muscles in the teat canal makes exotic breeds more susceptible to infection, and their udders are larger [63, 64]. However, the significance of genetic variation is often diminished by environmental factors. Overproduction of milk and management issues may be the reason for this discrepancy. High-producing exotic crossbreeds are also more prone to sick cow syndrome and mastitis infections due to their anatomical features [54]. In contrast to indigenous animals kept under an extensive system, alien animals in Ethiopia are frequently raised under a zero-grazing system with intensive management measures, which puts cows at risk for mastitis. Because alien animals produce more milk than native animals, their increased mastitis susceptibility is expected to make it difficult to increase host resistance to mastitis through breeding [65].

## 5. Conclusion

The pooled estimated prevalence of SCM in Ethiopian dairy cows was 43.19%. This review showed that the prevalence of SCM varies considerably by location. Age, parity, stage of lactation, and breed were found factors associated with SCM in dairy cows. The high prevalence of SCM could contribute to the low productivity of dairy cows in Ethiopia.

## Figures and Tables

**Table 1:** Summary of each research article included in the systematic review and meta-analysis of subclinical mastitis in dairy cows.

Authors	Region	Total sample size (N)	Diseased (n)	Prevalence (%)
[20]	Tigray	305	103	33.77
[21]	SNNP	183	56	30.60
[22]	Oromia	546	224	41.03
[23]	Oromia	90	66	73.33
[24]	Tigray	322	176	54.66
[25]	AA	365	164	44.93
[26]	Oromia	358	110	30.73
[27]	AA	444	302	68.02
[28]	Oromia	384	62	16.15
[29]	Oromia	151	48	31.79
[30]	SNNP	96	41	42.71
[5]	Oromia	262	105	40.08
[6]	Amhara	510	316	61.96
[8]	AA	390	192	49.23
[31]	Oromia	384	160	41.67
[32]	Oromia	290	137	47.24
[33]	Oromia	302	96	31.79
[34]	SNNP	138	71	51.45
[35]	Oromia	384	199	51.82
[36]	Oromia	384	92	23.96
[37]	Oromia	1049	346	32.98
[38]	Amhara	334	114	34.13
[39]	Oromia	384	242	63.02
[40]	Oromia	383	180	47.00
[10]	Oromia	384	172	44.79
[41]	SNNP	686	330	48.10
[42]	Tigray	146	56	38.36
[43]	Oromia	384	201	52.34
[2]	Oromia	532	121	22.74
[44]	Oromia	265	130	49.06
[45]	BG	367	104	28.34
[46]	Oromia	121	84	69.42
[9]	Amhara	328	197	60.06
[47]	SNNP	422	64	15.17

**Table 2:** A summary of each research article included in the systematic review and meta-analysis of subclinical mastitis in dairy cows.

Variable	Category	No. of studies	No. of examined	No. of positive	Prevalence (%)	Heterogeneity			
						95% Cl	*χ* ^2^	*p* value	*I* ^2^ (%)
Region	AA	3	1199	658	54.11	(40.18–68.03)	54.59	0.001	96.06
	Amhara	3	1172	627	52.07	(34.49–69.66)	77.06	0.001	97.47
	BG	1	367	104	28.34	(23.73–32.95)			
	Oromia	19	7037	2775	42.48	(35.64–49.32)	602.38	0.001	97.41
	SNNP	5	1525	562	37.37	(24.09–50.65)	189.69	0.001	96.69
	Tigray	3	773	335	41.34	(29.69–54.99)	30.60	0.001	92.27

Year	2012–2017	20	6288	2720	43.25	(36.90–49.59)	645.00	0.001	96.59
	2018–2022	14	5785	2341	43.11	(34.96–51.27)	590.61	0.001	97.79

Sample size	90–383	19	4806	2038	44	(38.16–49.84)	279.75	0.001	94.49
	384	7	2688	1128	41.91	(29.60–54.23)	349.84	0.001	98.00
	390–1049	8	4579	1895	42.36	(29.70–55.03)	609.48	0.001	98.88

Total		34	12,073	5061	43.19	(38.24–48.13)	1252.05	0.001	97.12

Abbreviations: AA = Addis Ababa, BG = Benishangul-Gumuz, SNNP = Southern nation, nationalities, and peoples.

**Table 3:** Assessment of publication bias using Egger's test.

Sta_Eff	Coefficient	Std. err.	*p*	(95% conf. interval)	
Slope	17.69177	9.253813	0.065	−1.15763	36.54117
Bias	9.358412	3.738803	0.018	1.74272	16.9741

*Note:* Sta_Eff = standard error of effect size, Std. err = standard error, and *p* = *p* value.

**Table 4:** Factors contributing to the estimated pooled prevalence of subclinical mastitis in dairy cows.

Factor	Category	No. of studies	No. of examined	No. of positive	Prevalence (95% CI)	Heterogeneity			Univariable metaregression	
						*χ* ^2^	*p* value	*I* ^2^ (%)	Coefficient (95% CI)	*p* value
Breed	Local	3	364	118	32 (19–44)	12.99	0.001	84.05	Ref.	
	Cross	3	500	233	49 (38–57)	10.8	0.001	79.24	16.3 (0.3–32.3)	0.046

Age	Young	5	994	228	34 (27–441)	18.09	0.001	79.96	Ref.	
	Adult	4	492	215	43 (37–50)	5.76	0.130	47.36	9.0 (−1.73–19.73)	0.100
	Old	4	186	119	64 (52–76)	9.14	0.030	65.37	30.29 (18.3–42.28)	0.001

Parity	1–3	3	635	222	35 (28–42)	6.88	0.030	71.24	Ref.	
	4–7	3	270	149	54 (34–75)	28.6	0.001	91.91	19.1 (−2.0–40.2)	0.076
	> 7	1	17	15	88 (74–104)				53.1 (20.9–85.4)	0.001

Lactation stage	Early	4	703	196	27.5 (11.04–43.96)	158.5	0.001	96.8	Ref.	
	Mid	4	422	165	38.7 (28.51–48.89)	17.07	0.001	78.68	11.69 (−5.36–28.37)	0.179
	Late	4	285	150	52.67 (46.9–58.45)	2.41	0.49	0.00	25.42 (7.96–42.87)	0.004

Management system	Intensive	2	258	126	46 (41–56)	1.35	0.240	26.09	Ref.	
	Extensive	1	39	14	36 (21–51)				−11.9 (−63.3–39.5)	0.650
	Semi-intensive	2	185	39	38 (−6–81)	14.01	0.001	92.86	−11.7 (−53.4–30.0)	0.583

*Note:* Ref = reference, *I*^2^ = inverse variance index, *χ*^2^ = Chi-square.

Abbreviation: CI = confidence interval.

## Data Availability

The data that were extracted, processed, and analyzed for this work are available from the corresponding author upon reasonable request.
